# Distinct roles of exogenous opioid agonists and endogenous opioid peptides in the peripheral control of neuropathy-triggered heat pain

**DOI:** 10.1038/srep32799

**Published:** 2016-09-08

**Authors:** Dominika Labuz, Melih Ö. Celik, Andreas Zimmer, Halina Machelska

**Affiliations:** 1 Department of Anesthesiology and Critical Care Medicine, Charité-Universitätsmedizin Berlin, Campus Benjamin Franklin, Hindenburgdamm 30, 12203 Berlin, Germany; 2 Institute of Molecular Psychiatry, University of Bonn, 53105 Bonn, Germany

## Abstract

Neuropathic pain often results from peripheral nerve damage, which can involve immune response. Local leukocyte-derived opioid peptides or exogenous opioid agonists inhibit neuropathy-induced mechanical hypersensitivity in animal models. Since neuropathic pain can also be augmented by heat, in this study we investigated the role of opioids in the modulation of neuropathy-evoked heat hypersensitivity. We used a chronic constriction injury of the sciatic nerve in wild-type and opioid peptide-knockout mice, and tested opioid effects in heat and mechanical hypersensitivity using Hargreaves and von Frey tests, respectively. We found that although perineural exogenous opioid agonists, including peptidergic ligands, were effective, the endogenous opioid peptides β-endorphin, Met-enkephalin and dynorphin A did not alleviate heat hypersensitivity. Specifically, corticotropin-releasing factor, an agent triggering opioid peptide secretion from leukocytes, applied perineurally did not attenuate heat hypersensitivity in wild-type mice. Exogenous opioids, also shown to release opioid peptides via activation of leukocyte opioid receptors, were equally analgesic in wild-type and opioid peptide-knockout mice, indicating that endogenous opioids do not contribute to exogenous opioid analgesia in heat hypersensitivity. Furthermore, exogenously applied opioid peptides were ineffective as well. Conversely, opioid peptides relieved mechanical hypersensitivity. Thus, both opioid type and sensory modality may determine the outcome of neuropathic pain treatment.

Pain is the most common reason for medical visits. It is a health problem of the high socio-economic relevance, with approximately 20% of adults suffering chronic pain globally^1^. Neuropathic pain can develop following peripheral nerve lesions (e.g., surgery, compression, entrapment), it decreases the quality of life and is difficult to treat^1^,^2^. Nonsteroidal anti-inflammatory drugs are usually ineffective, while the use of antidepressants and anticonvulsants is limited by nausea, gait disturbance and risk of cardiovascular complications. Opioids such as morphine were reported efficacious in some neuropathic conditions; however, adverse effects such as nausea, apnoea, sedation and addiction resulting from the actions in the central nervous system (CNS), often hinder the therapy^2^,^3^.

Apart from the CNS, opioid receptors (μ, δ and κ) are also expressed in peripheral sensory neurones. Peripheral opioid receptors considerably contribute to the analgesic effects of opioid drugs applied systemically in animal inflammatory and neuropathic pain models, and in postoperative pain in humans^4^,^5^,^6^,^7^,^8^. There is also substantial evidence that selective activation of peripheral opioid receptors by injections of exogenous opioids into injured tissue results in attenuation of neuropathy-induced mechanical and heat hypersensitivity in animal models^9^,^10^,^11^,^12^,^13^,^14^.

Additionally, opioid receptor endogenous ligands, i.e., opioid peptides such as β-endorphin (END), Met-enkephalin (ENK) and dynorphin A 1–17 (DYN) are synthesized and secreted by immune cells in response to stressful stimuli (e.g., experimental swim stress, surgery) or stimulation by corticotropin-releasing factor (CRF), formyl peptides and chemokines (CXCL2/3). The released opioid peptides activate peripheral neuronal opioid receptors resulting in amelioration of experimental and clinical inflammatory pain^5^,^15^,^16^,^17^. Majority of studies reported improvement of mechanical hypersensitivity by leukocyte-derived opioids^18^,^19^,^20^,^21^,^22^,^23^,^24^,^25^,^26^, while few showed attenuation of heat hypersensitivity in inflammatory pain^23^,^24^.

Interestingly, many studies revealed that not only somatic inflammatory pain but also neuropathy can be associated with immune reactions. Nerve injuries lead to the activation of resident cells (e.g., macrophages, Schwann cells, fibroblasts) and influx of blood-born leukocytes, including neutrophils, monocytes/macrophages and T lymphocytes. By secreting inflammatory cytokines (e.g., interleukin-1β, tumor necrosis factor-α) these cells can contribute to the generation of neuropathic pain^5^,^27^,^28^. On the other hand, immune cells accumulating at the damaged nerves also contain opioid peptides^29^,^30^,^31^,^32^,^33^, and can ameliorate neuropathy-induced tactile hypersensitivity. Hence, selective stimulation of opioid-cells by CRF^29^,^30^ or activation of opioid receptors in leukocytes by exogenous opioid agonists^33^, which leads to the secretion of opioid peptides^20^,^33^, abolished mechanical hypersensitivity in a chronic constriction injury (CCI) neuropathic pain model^29^,^30^,^33^.

Neuropathic pain can also be amplified by heat stimuli^2^,^34^. Therefore, in this study we aimed to evaluate the role of endogenous opioid peptides in the peripheral regulation of neuropathy-evoked heat hypersensitivity following CCI of the sciatic nerve in mice. Intriguingly, we found that although exogenous opioid agonists applied perineurally at the injured nerves ameliorated heat hypersensitivity, the opioid peptides did not. This is in contrast to mechanical hypersensitivity, which was improved by opioid peptides. These findings suggest that opioid therapy may require careful tailoring according to the opioid type and modality of pain resulting from neuropathy.

## Results

### Effects in response to CRF

CRF is an agent releasing opioid peptides from leukocytes and thereby elicits analgesia in peripheral inflamed tissue^16^,^20^,^35^. Previously we have shown in a mouse CCI neuropathic pain model that in response to CRF perineural application at the injured nerve (CCI site), the opioid peptides derived from locally accumulating immune cells attenuate mechanical hypersensitivity evaluated using von Frey filaments. This conclusion was based on the findings that CRF-induced analgesia was abolished by both leukocyte depletion and co-application of opioid receptor antagonists or antibodies to opioid peptides^29^.

**Figure 1:**
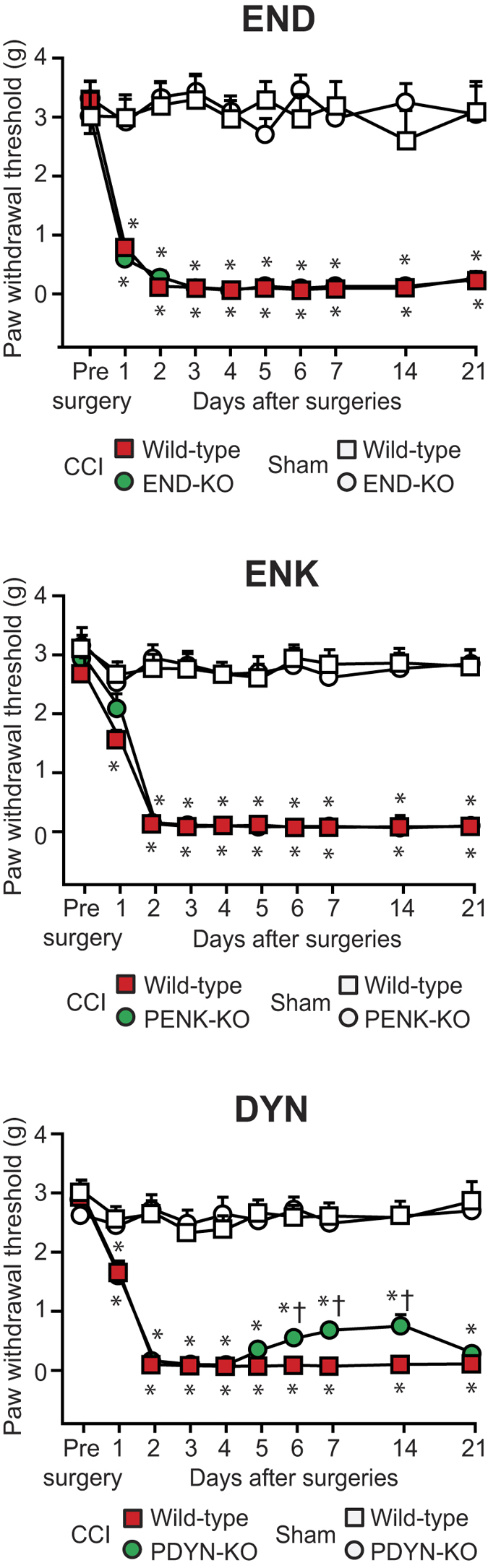
Development of mechanical hypersensitivity in wild-type and opioid peptide-lacking mice. The effects were measured using von Frey filaments in hind paws ipsilateral to the CCI or sham surgery in wild-type, END-KO, PENK-KO or PDYN-KO mice. *P < 0.05 compared to the corresponding thresholds before CCI surgery and to those in sham-operated mice; ^†^P < 0.05 compared to the corresponding wild-type mice (two-way repeated measures ANOVA, Bonferroni test). There were no significant differences in sham-operated groups (P > 0.05, two-way repeated measures ANOVA). Detailed statistical analysis is presented in Supplementary Table 1. Data are expressed as mean ± SEM. N = 6–8 mice per group.

In this study, to verify the effect of CRF on mechanical hypersensitivity, we used mice lacking opioid peptides. These were mice with a point mutation in the proopiomelanocortin (POMC) gene selectively lacking the region encoding END, here referred to as END-knockout (KO)^36^, and mice lacking the ENK precursor proenkephalin (PENK-KO)^37^ or the DYN precursor prodynorphin (PDYN-KO)^38^. Following CCI, wild-type, END-KO and PENK-KO mice developed comparable mechanical hypersensitivity, which was demonstrated by significantly lower von Frey thresholds in paws innervated by injured nerves as compared to the thresholds before surgery and to the thresholds of sham-operated mice. This hypersensitivity appeared on day 1 or 2 and lasted until day 21 following CCI (Fig. 1). In PDYN-KO mice the mechanical thresholds were slightly, but significantly higher than in wild-type mice on days 6–14 after CCI (Fig. 1). There were no significant alterations in the thresholds of paws innervated by sham-operated nerves (Fig. 1) and in the thresholds of paws contralateral to CCI or sham surgery (P > 0.05; data not shown).

**Figure 2:**
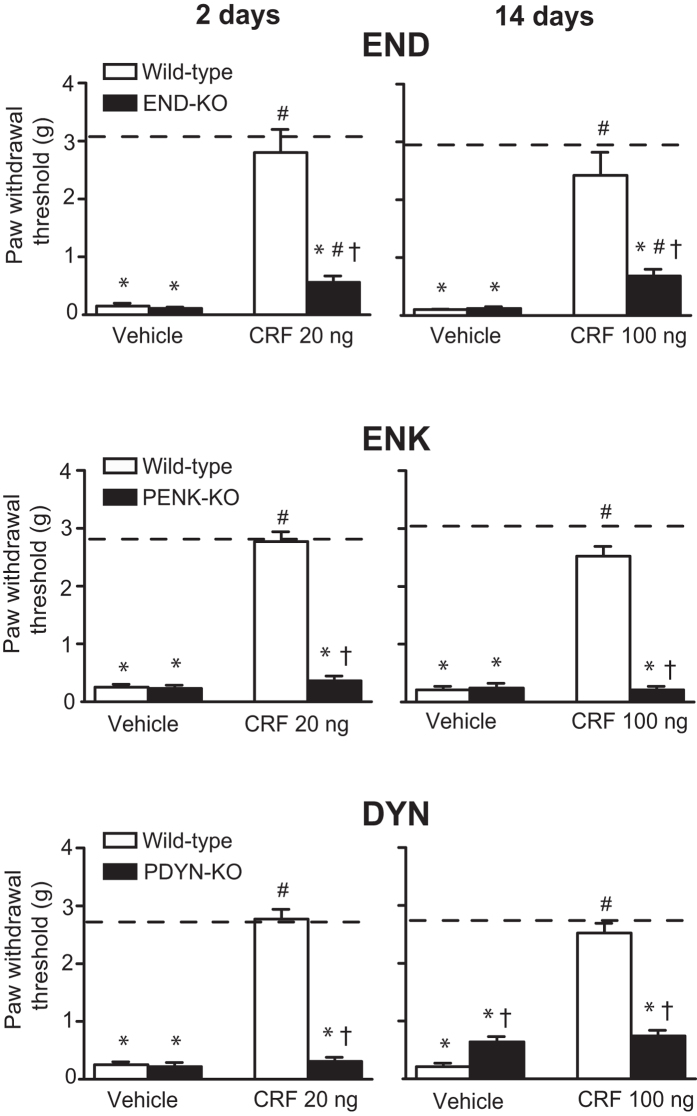
Effects of CRF on mechanical hypersensitivity in wild-type and opioid peptide-lacking mice. CFR was injected at the CCI site on days 2 and 14 following CCI, and effects were assessed 30 min after injection using von Frey filaments in hind paws ipsilateral to the CCI of wild-type, END-KO, PENK-KO or PDYN-KO mice. *P < 0.05 compared to thresholds before CCI (indicated by dashed lines) (paired t-test or Wilcoxon test); ^#^P < 0.05, compared to the respective vehicle-treated groups; ^†^P < 0.05 compared to the corresponding wild-type mice (Mann-Whitney U test). Data are expressed as mean ± SEM. N = 6–8 mice per group.

CRF applied at the CCI site in the most effective doses (20 ng at 2 days or 100 ng at 14 days) found earlier^29^,^30^, significantly elevated von Frey thresholds, completely reversing mechanical hypersensitivity (to the thresholds before CCI) measured 30 min after injection, on days 2 and 14 following CCI in wild-type mice. In contrast, these CRF analgesic effects were virtually absent in END-KO, PENK-KO and PDYN-KO mice (Fig. 2). Vehicle had no effect (Fig. 2) and there were no significant changes in contralateral paws in all genotypes (P > 0.05; data not shown). These results confirm that in response to CRF, all three opioid peptides, END, ENK and DYN at the site of nerve injury ameliorate neuropathy-induced mechanical hypersensitivity.

**Figure 3:**
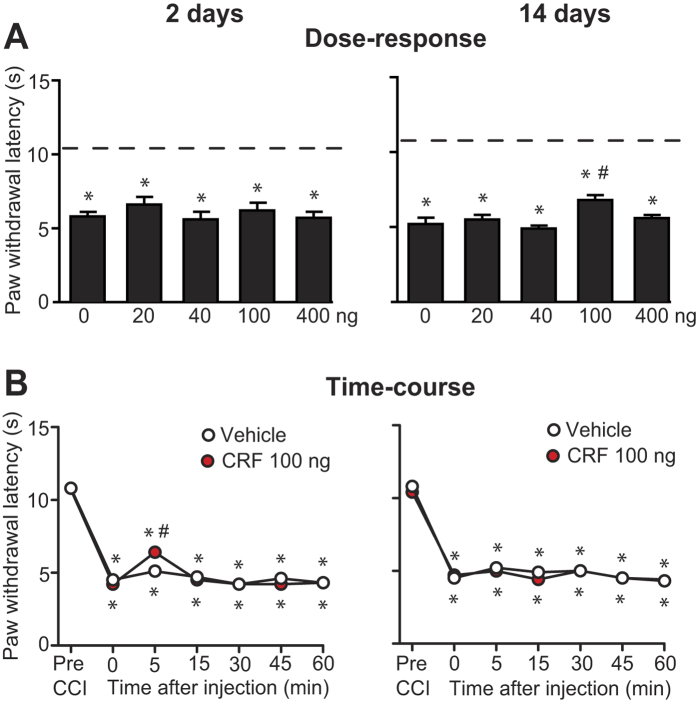
Effects of CRF on heat hypersensitivity in wild-type mice. (**A**) Dose-dependency assessed 5 min after CFR injection. *P < 0.05 compared to latencies before CCI (indicated by dashed lines) (paired t-test or Wilcoxon test); ^#^P < 0.05 compared to vehicle-treated group (0 ng) (one-way ANOVA, Bonferroni test). (**B**) Time-course of CFR (100 ng) effects. *P < 0.05 compared to latencies before CCI; ^#^P < 0.05, compared to vehicle-treated group (two-way repeated measures ANOVA, Bonferroni test). In all experiments, CRF was applied at the CCI site and the effects were assessed using Hargreaves test, in hind paws ipsilateral to the CCI on days 2 and 14. Detailed statistical evaluation is presented in Supplementary Table 2. Data are expressed as mean ± SEM. N = 6 mice per group.

However, when tested in hypersensitivity to heat (using Hargreaves test), which was demonstrated by significantly shortened withdrawal latencies in paws innervated by injured nerves (Fig. 3), the CRF applied at the CCI site in a wide dose range (20–400 ng) and tested up to 60 min following injections, was ineffective in wild-type mice. Although occasionally there were some statistically significant elevations in paw withdrawal latency (on day 14 when tested for dose-dependency, and on day 2 when tested for time-course), these effects are very small and substantially below the latency measured before CCI, and unlikely to be of biological relevance (Fig. 3). Since CRF was ineffective in wild-type mice, there was no rationale to test it in KO mice in heat hypersensitivity.

### Effects in response to exogenous opioid receptor agonists

Neuropathy-induced mechanical hypersensitivity can also be ameliorated by opioid peptides derived from immune cells upon activation of opioid receptors. Hence, activation of leukocyte μ-, δ- and κ-opioid receptors by selective exogenous agonists triggered the intracellular Ca^2+^-regulated opioid peptide release and resulted in analgesia. This statement was supported by the findings that exogenous agonists applied at the CCI site (infiltrated by opioid peptide-containing leukocytes) ameliorated mechanical hypersensitivity, which was attenuated by immune cell depletion or by genetic deletion of opioid peptides in KO mice^33^.

**Figure 4:**
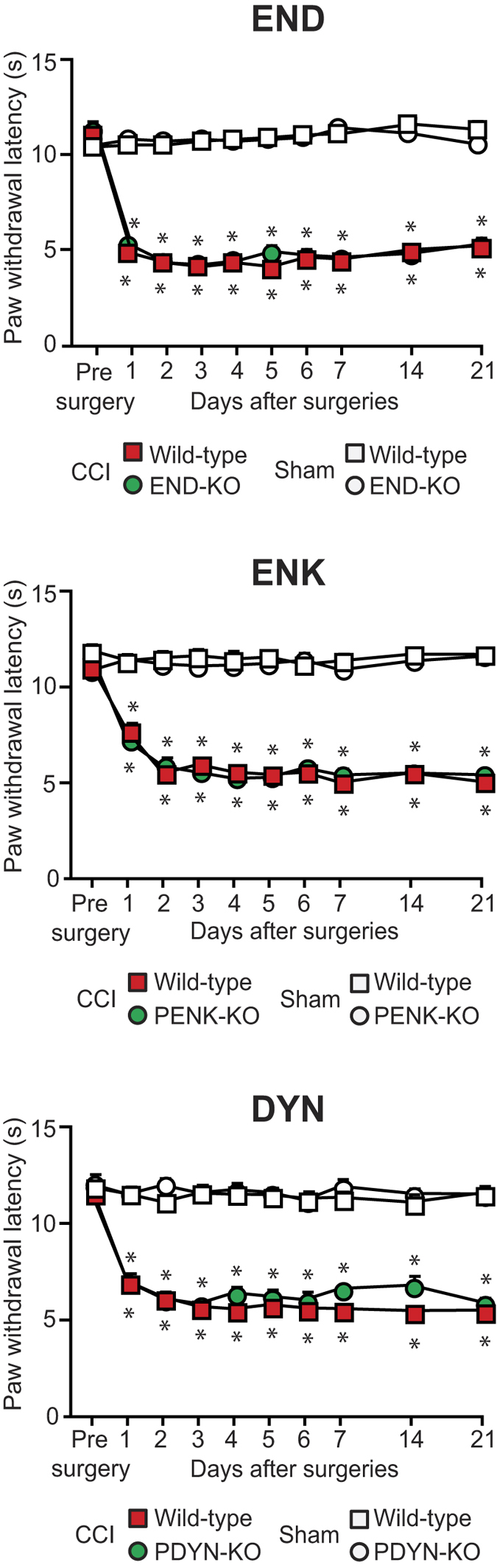
Development of heat hypersensitivity in wild-type and opioid peptide-lacking mice. The effects were measured using the Hargreaves test in hind paws ipsilateral to the CCI or sham surgery in wild-type, END-KO, PENK-KO or PDYN-KO mice. *P < 0.05 compared to the corresponding latencies before CCI surgery and to those in sham-operated mice (two-way repeated measures ANOVA, Bonferroni test). There were no significant differences in sham-operated groups (P > 0.05, two-way repeated measures ANOVA). Detailed statistical analysis is presented in Supplementary Table 3. Data are expressed as mean ± SEM. N = 6–8 mice per group.

In this study, to examine whether endogenous opioid peptides contribute to exogenous agonist-induced analgesia in heat hypersensitivity following neuropathy, we evaluated exogenous agonist analgesia in opioid peptide-KO mice using Hargreaves test. Following CCI, all mice, including wild-type, END-KO, PENK-KO and PDYN-KO developed comparable heat hypersensitivity in paws innervated by injured nerves as compared to paws of sham-operated mice, on days 1–21 (Fig. 4). There were no significant alterations in the withdrawal latencies of paws innervated by sham-operated nerves (Fig. 4) and in the latencies of paws contralateral to CCI or sham surgery (P > 0.05; data not shown).

**Figure 5:**
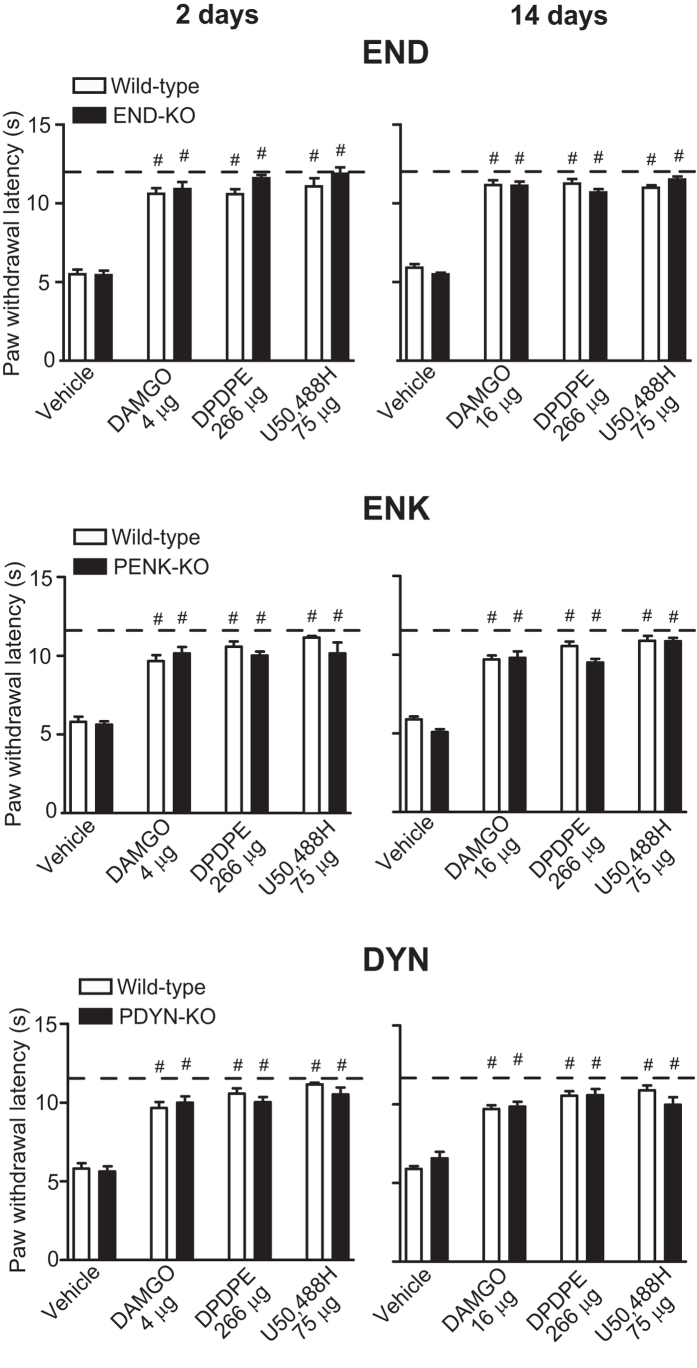
Effects of exogenous opioid receptor agonists on heat hypersensitivity in wild-type and opioid peptide-lacking mice. DAMGO, DPDPE and U50, 488H were injected at the CCI site on days 2 and 14 following CCI, and effects were assessed 5 min after injection using Hargreaves test, in hind paws ipsilateral to the CCI of wild-type, END-KO, PENK-KO or PDYN-KO mice. Dashed lines represent latencies determined before CCI. ^#^P < 0.05 compared to the corresponding vehicle-treated groups (two-way ANOVA, Bonferroni test). Detailed statistical evaluation is presented in Supplementary Table 4. Data are expressed as mean ± SEM. N = 8–9 mice per group.

To examine exogenous opioid analgesia we used selective agonists of μ-opioid receptors DAMGO ([D-Ala2, N-Me-Phe4, Gly5-ol]-enkephalin; 4 μg on day 2 and 16 μg on day 14), δ-opioid receptors DPDPE (D-Pen2, D-Pen5-enkpephalin; 266 μg) and κ-opioid receptors U50, 488H (trans-(6)3,4-dichloro-N-methyl-N-[2-(1-pyrrolidinyl)-cyclohexyl]-benzeneacetamide; 75 μg). All agonists applied at the CCI site in the most effective doses^14^ fully reversed heat hypersensitivity, reaching the latencies measured before CCI in wild-type mice on days 2 and 14 following CCI (Fig. 5). However, similar effects were produced in all three opioid peptide-KO lines (Fig. 5), indicating that endogenous opioid peptides do not contribute to analgesia produced by exogenous opioid agonists in heat hypersensitivity.

### Effects of opioid peptides

**Figure 6:**
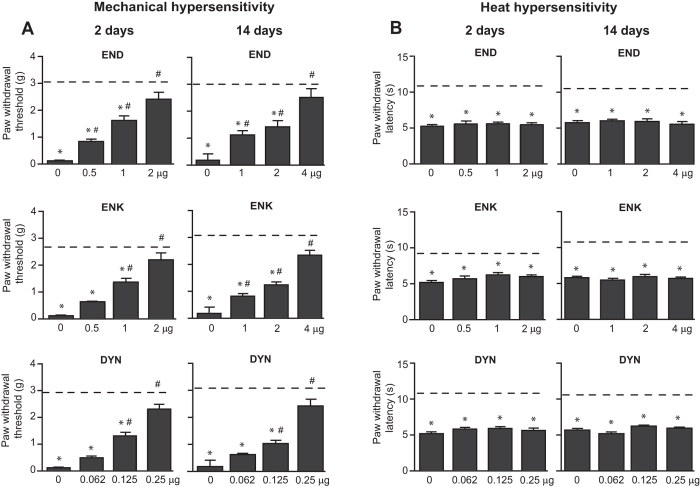
Effects of exogenously applied opioid peptides on mechanical and heat hypersensitivity in wild-type mice. (**A**) Effects of opioid peptides on mechanical hypersensitivity assessed using von Frey filaments. (**B**) Effects of opioid peptides on heat hypersensitivity measured using Hargreaves test. In all experiments, opioid peptides were applied at the CCI site and the effects were assessed 5 min later, in hind paws ipsilateral to the CCI on days 2 and 14. *P < 0.05 compared to thresholds or latencies before CCI (indicated by dashed lines) (paired t-test or Wilcoxon test); ^#^P < 0.05 compared to vehicle-treated group (0 μg) (one-way ANOVA, Bonferroni test). Detailed statistical analysis is presented in Supplementary Table 5. Data are expressed as mean ± SEM. N = 7–8 mice per group.

The results described above suggest that although exogenous agonists, including peptidergic DAMGO and DPDPE are effective, the endogenous opioid peptides do not attenuate neuropathy-induced heat hypersensitivity. Therefore, in the following experiments we assessed whether these effects can be mimicked by exogenously applied opioid peptides and whether they are limited to heat hypersensitivity by also testing them in mechanical hypersensitivity. Indeed, we found that END (0.5–4 μg), ENK (0.5–4 μg) and DYN (0.0625–0.25 μg) applied at the CCI site dose-dependently reversed mechanical hypersensitivity (Fig. 6A), but did not attenuate heat hypersensitivity neither on day 2 nor 14 following CCI (Fig. 6B).

## Discussion

The main finding in this study is that although peripherally acting exogenous opioid receptor agonists are effective, the endogenous opioid peptides do not alleviate neuropathy-induced heat hypersensitivity. In contrast, mechanical hypersensitivity can be ameliorated by both exogenous agonists^14^,^33^ and endogenous opioid peptides (this study).

These differences apply to the effects of both CRF (Fig. 3) and exogenous opioid agonists (Fig. 5), which can induce secretion of opioid peptides from immune cells^20^,^33^,^35^. To examine whether these effects are restricted to heat hypersensitivity, we first verified the action of CRF in mechanical hypersensitivity using opioid peptide-deficient mice. When tested over time, PDYN-KO mice developed slightly weaker neuropathy-induced mechanical hypersensitivity compared to wild-type animals (Fig. 1), suggesting some pro-nociceptive tone of endogenous DYN. This observation is in line with previous studies proposing that these effects mostly occur at the spinal cord level and can be mediated by both κ-opioid receptors and N-methyl D-aspartate receptors^39^,^40^. In opposite to PDYN-KO, mice lacking END or PENK developed comparable hypersensitivity to wild-type mice (Figs 1 and 4), indicating no major contribution of END and ENK to the tonic control of neuropathic pain. PENK-KO mice have not been previously examined following nerve injury, but the findings in END-KO mice are in line with an earlier report^41^. Importantly, we found that amelioration of mechanical hypersensitivity following perineural CRF application observed in wild-type mice was absent in all three opioid peptide-KO lines (Fig. 2), in accordance with the blockade of CRF analgesia by opioid peptide antibodies, found previously^29^. Clearly, however, CRF did not improve heat hypersensitivity (Fig. 3). This result somewhat differs from the data showing that neuropathy-induced heat hypersensitivity was diminished by opioid peptide-containing leukocytes recruited by granulocyte colony stimulating factor (G-CSF)^32^. As these discrepancies might relate to the stimulating factor type (CRF vs. G-CSF), the actions of G-CSF appear complex, since other studies found this growth factor did not improve inflammatory pain^42^ or even exacerbated neuropathic pain^43^.

Recently we found that inhibition of CCI-induced mechanical hypersensitivity produced by exogenous opioid agonists applied perineurally was diminished by 40–70% in opioid peptide-KO mice compared to wild-type mice^33^. Conversely, the endogenous opioid peptides at damaged nerves do not seem to contribute to local exogenous opioid agonist-induced attenuation of heat hypersensitivity (Fig. 5). In the previous study we have confirmed the selectivity of DAMGO to μ-opioid receptors, DPDPE to δ-opioid receptors, and U50, 488H to κ-opioid receptors by showing that analgesia induced by each agonist was only blocked by the respective receptor antagonist^14^. Additionally, other studies employing mice lacking either μ-, δ- or κ-opioid receptors verified the respective agonist selectivity in various assays, including receptor binding, respiratory depression, analgesia or analgesic tolerance (reviewed in ref. 44). On the other hand, just as a possible lack of specificity of pharmacological tools, compensatory changes in KO animals are also a potential drawback. In opioid peptide-KO mice, no changes in the expression of remaining opioid peptides were observed. There were also no alterations in the expression of opioid receptors in PDYN-KO and END-KO mice, but μ- and δ-binding sites were upregulated in PENK-KO mice, in brain regions associated with emotional behaviours; not in pain-related regions though (reviewed in ref. 44). Even if such changes occurred in the peripheral pain pathways, they do not seem to play a major role in our study because all three μ-, δ- and κ-opioid receptor agonists exerted comparable analgesic effects not only in PENK-KO but also in END-KO and PDYN-KO mice. In fact, the exogenous opioid analgesia did not differ between wild-type and KO mice (Fig. 5), and thus any potential compensatory changes in KO mice do not appear to be of relevance in our experimental conditions.

Our findings are in agreement with previous work evaluating exogenous opioid analgesia in opioid peptide-deficient mice following systemic, spinal or intracerebroventricular opioid agonist applications, in heat hypersensitivity. Indeed, in most of those studies the exogenous analgesia was unaltered in KO mice compared to wild-type mice under healthy conditions (without tissue damage) or in an inflammatory pain model^38^,^45^,^46^,^47^. In a partial sciatic nerve ligation-induced neuropathy, systemically applied morphine and U50, 488H (in the most effective doses) in END-KO and PDYN-KO mice, respectively, also produced comparable analgesia to that in wild-type mice^40^,^41^; however, mechanical hypersensitivity was not examined. In the early 1980s, spinally or intracerebroventricularly applied END, ENK and DYN were reported to attenuate tail pinch and tail-flick responses in naïve (without tissue injury) rats or mice (reviewed in ref. 48). More recently, spinal delivery of vectors encoding POMC or PENK improved both mechanical and heat hypersensitivity following CCI^49^,^50^; yet, it is unclear whether actions of such genetically delivered and native opioid peptides are mechanistically identical. Together, the relative contribution of the CNS endogenous opioids to mechanical vs. heat hypersensitivity in neuropathic conditions has not been systematically examined.

Our study suggests that distinct roles of exogenous opioid agonists and endogenous opioid peptides in the peripheral control of neuropathic pain predominantly apply to heat hypersensitivity. This finding is supported by experiments showing that also exogenously applied END, ENK and DYN fully reversed mechanical hypersensitivity, but were ineffective in heat hypersensitivity (Fig. 6). This was somewhat unexpected, since both exogenous and endogenous opioids are agonists of opioid receptors, which are expressed in C and Aδ afferents, and in a lower degree in Aβ fibres^6^,^7^,^51^, and these afferents respond to mechanical and thermal stimulation^52^. It is thus possible that exogenous and endogenous opioids differently interact with transducer molecules sensing mechanical stimuli (e.g., potassium channels) or heat (e.g., transient receptor potential channels)^53^. DAMGO decreased capsaicin-induced activity of transient receptor potential vanilloid 1 (TRPV1) in dorsal root ganglion nociceptors assessed by patch clamp^54^, but DPDPE, U50, 488H and opioid peptides, and their effects in response to heat stimulation have not been examined. On the other hand, TRPV1 may not be the best candidate, since its blockade can attenuate both heat and mechanical hypersensitivity in pathological pain models (ref. 55 and refs. therein). A biased agonism (or functional selectivity) might still be an option. This phenomenon is characterised by the ability of different ligands of the same receptor to stabilise various receptor active states leading to the activation of diverse signalling pathways. This concept has been mostly examined for exogenous opioid receptor agonists and only few studies addressed opioid peptides. For example, in AtT20 mouse pituitary tumour cell line expressing mouse μ-opioid receptors, compared to DAMGO, ENK showed bias towards μ-receptor internalization over inhibition of cyclic adenosine monophosphate formation, and towards cell hyperpolarization over β-arrestin recruitment^56^. Nonetheless, whether such effects translate to *in vivo* conditions and how they correspond to the modulation of mechanical vs. heat hypersensitivity by opioids is very challenging to examine and remains speculative. Interestingly, however, in a spinal nerve injury-induced neuropathy, μ-opioid receptor-KO mice had increased responses to mechanical but not heat stimulation, whereas in WT mice morphine attenuated both hypersensitivity forms^57^. Analogously, conditional deletion of δ-opioid receptors in Nav1.8 nociceptors resulted in enhanced mechanical but not heat hypersensitivity, while δ-opioid receptor agonist SNC80 attenuated both hypersensitivity types in WT mice in a partial sciatic nerve ligation neuropathic pain model^6^. Hence, the findings of both studies suggest that exogenous μ- and δ-agonists attenuate responses to mechanical and heat stimuli, while their endogenous agonists control mechanical but not heat hypersensitivity following neuropathy, in accord with our data. Another study reported that DYN applied into paws innervated by injured nerves following CCI attenuated both hypersensitivity types; however, not all data were shown, the effects appeared much weaker in heat hypersensitivity, and the use of rats^11^ vs. mice (this study) might be additional factor contributing to these differing results.

Currently it is uncertain how these effects apply to other pain models. For example, in a complete Freund’s adjuvant-induced hind paw inflammation in rats, opioid peptides secreted from immune cells by chemokine CXCL2/3 or formyl peptides attenuated both mechanical and heat hypersensitivity^23^,^24^. CRF as well as END, ENK and DYN injected into inflamed paws improved mechanical hyperalgesia, but they were not tested in heat hypersensitivity^18^,^19^,^20^,^21^,^22^,^58^,^59^,^60^. Thus, it remains to be elucidated whether the type of opioid peptide-releasing factor, animal species and/or pain model determine possible different roles of exogenous and endogenous opioids in the modulation of heat hypersensitivity.

In conclusion, our data suggest the differences between synthetic opioids (DAMGO, DPDPE, U50, 488H) and native opioid peptides (END, ENK, DYN) in the regulation of neuropathic pain. Interestingly, even though DAMGO and DPDPE are derivatives of ENK, only exogenous opioid agonists but not endogenous opioid peptides ameliorated heat pain. While the reasons for the intriguing finding that these effects apply to neuropathy-triggered heat but not mechanical hypersensitivity need to be explored, it appears that both opioid type and modality of pain resulting from nerve damage might determine the successful therapy. These findings may be helpful considerations in the view of an interest in the development of peptide-based pain medications^11^,^61^.

## Methods

### Animals

Experiments were approved by the State animal care committee (Landesamt für Gesundheit und Soziales, Berlin, Germany) and were performed according to the ARRIVE guidelines^62^. The animals were male mice (22–30 g, 6–13 weeks old), either wild-type (C57BL/6J; Harlan Laboratories) or lacking END, PENK or PDYN. PENK-KO and PDYN-KO mice backcrossed to C57BL/6J background for at least ten generations were provided by A. Zimmer^37^,^38^. The heterozygous END^+/−^ mice (on C57BL/6J background) were purchased from the Jackson Laboratory (B6.129S2-*Pomc*^*tm1Low*^/J; stock number 00319)^36^ and bred with heterozygotes or wild-type (C57BL/6J) mice to obtain END-KO homozygotes. Since all KO mice were backcrossed to C57BL/6J background for at least ten generations, non-littermate C57BL/6J wild-type mice were used as controls. All animals were bred at the Charité, Berlin, and were kept in groups of 3–5 per cage, with free access to food and water, in environmentally controlled conditions (12 h light/dark schedule, light on at 7:00 h; 22 ± 0.5 °C; humidity 60–65%).

Animals were randomly placed in cages by an animal caretaker not involved in the study. Experiments were blinded regarding the genotypes and treatments/doses. Substances were prepared in separate, coded vials by a colleague not involved in *in vivo* testing. The codes were broken after completion of experiments. No statistical test was run to determine sample size a priori; the animal numbers are similar to those used in previous studies^6^,^10^,^11^,^14^,^29^,^55^. Each group consisted of 6–9 animals and was tested on 2–3 different days. After completion of experiments, animals were killed with isoflurane overdose (AbbVie, Ludwigshafen, Germany). All efforts were made to minimize animal numbers and suffering.

### PCR

Mice were genotyped by PCR, as described previously^33^. Briefly, ear tissue punches were digested overnight and genomic DNA was purified. To genotype PENK-KO and PDYN-KO mice the following primers were used: PENK (forward 5′-GCATCCAGGTAATTGGCAGGAA-3′, reverse 5′-CAGCAGCCTCTGTTCCACATACACTTCAT-3′, middle 5′-TCCTTCACATTCCAGTGTGC-3′), PDYN (forward 5′-CGCACCGTCCATTTTAATGAGGAGGACTTG-3′, reverse 5′-CTTCAGAATAGGTATTGGGGTTCTCCTGGG-3′, middle 5′-AGCGCATCGCCTCTCATCGCCTTCTT-3′). To genotype END-KO mice, two primer sets were used; one set (forward 5′-GAAGTACGTCATGGGTCACT-3′, reverse 5′-GACATGTTCATCTCTATACATAC-3′) amplified PCR products corresponding to the wild-type POMC, and a second set (forward 5′-GAGGATTGGGAAGACAATAGCA-3, reverse 5′-GACATGTTCATCTCTATACATAC-3′) amplified PCR products corresponding to the truncated POMC. All primers were purchased from TIBMOLBIOL (Berlin, Germany).

### Neuropathy

CCI was induced in deeply isoflurane-anesthetized mice by exposing the sciatic nerve at the level of the right mid-thigh and placing three loose silk ligatures (4/0) around the nerve with about 1-mm spacing; the ligatures were tied until they elicited a brief twitch in the respective hind limb. The wound was closed with silk sutures. Sham operation was performed in a similar manner but without nerve ligation^14^,^29^,^30^,^33^.

### Assessment of nociception

In all experiments, animals were habituated to the test cages daily (1–2 times for 15 min), starting 6 days prior to nociceptive testing. During the testing, the sequence of paws was alternated between animals to avoid “order” effects.

#### Mechanical sensitivity (von Frey test)

Animals were individually placed in clear Plexiglas cubicles located on a stand with anodized mesh (Model 410; IITC Life Sciences, Woodland Hills, CA). The calibrated von Frey filaments in the range of 0.054 mN (0.0056 g) to 42.85 mN (4.37 g) were used (Stoelting, Wood Dale, IL). The filaments were applied until they bowed, for approximately 3 s, to the plantar surface of hind paws. The up-down method was used to estimate 50% withdrawal thresholds^63^. Testing began using a 2.74 mN (0.28 g) filament. If the animal withdrew the paw, the just preceding weaker filament was applied. In the case of no withdrawal, the next stronger filament was applied. The maximal number of applications was 6–9, and the cut-off was 42.85 mN (4.37 g), according to our previous studies^14^,^29^,^30^,^33^.

#### Heat sensitivity (Hargreaves test)

Mice were individually placed in clear Plexiglas chambers positioned on a stand with glass surface (Model 336; IITC Life Sciences, Woodland Hills, CA). Radiant heat was applied to the plantar surface of hind paws from underneath the glass floor with a high-intensity projector lamp bulb and paw withdrawal latency was evaluated using an electronic timer. The withdrawal latency was defined as the average of two measurements separated by at least 10 s. The heat intensity was adjusted to obtain baseline withdrawal latency of about 10–12 s in uninjured paws, and the cut-off was 20 s to avoid tissue damage^14^,^55^.

### Substances

The following substances were used: CRF, U50, 488H (Sigma Aldrich, Deisenhofen, Germany), DAMGO, DPDPE, END, ENK (Bachem, Weil am Rhein, Germany), and DYN (Tocris, Wiesbaden-Nordenstadt, Germany). All substances were dissolved in sterile water to obtain stock solutions and diluted with 0.9% NaCl. Control groups were treated with 0.9% NaCl.

All substances were injected near the nerve at the CCI site (30 μl) under brief isoflurane anaesthesia. A polyethylene tube was placed 2 mm from the tip around the 26G needle to ensure the same depth of needle insertion into the middle of the scar tissue after operation^14^,^29^,^30^,^33^.

### Experimental protocols

The development of mechanical and heat sensitivity was evaluated a day before and daily on days 1–7, 14 and 21 following CCI or sham surgery in wild-type and opioid peptide-KO mice. The effects of CRF and opioids on mechanical and heat hypersensitivity were assessed on days 2 and 14 following CCI. The effects of CRF on mechanical hypersensitivity (20 ng on day 2; 100 ng on day 14) were measured 30 min after CRF injection, in wild-type and opioid peptide-KO mice. The dose-dependency of CRF (20–400 ng) on heat hypersensitivity was tested at 5 min, while the time-course of CRF (100 ng) was evaluated before and 5–60 min following injection in wild-type mice. The contribution of endogenous opioid peptides to exogenous opioid analgesia in heat hypersensitivity was examined by testing effects of DAMGO (4 μg on day 2; 16 μg on day 14), DPDPE (266 μg), and U50, 488H (75 μg) at 5 min following injections, in wild type and opioid peptide-KO mice. The effects of END (0.5–4 μg), ENK (0.5–4 μg) and DYN (0.0625–0.25 μg) were assessed 5 min following injections. Control groups were examined accordingly. Doses and time-course are based on our pilot experiments and earlier studies^14^,^29^,^30^.

### Statistical analysis

The data are expressed as means ± SEM. Two-sample comparisons were performed using paired t-test for dependent normally-distributed data, Wilcoxon test for dependent not-normally-distributed data, and Mann-Whitney U test for independent not-normally-distributed data. Dose-response relationships were analysed by one-way analysis of variance (ANOVA) followed by Bonferroni test. Two-way repeated measures ANOVA followed by Bonferroni test was used to compare two treatments over time. Two-way ANOVA followed by Bonferroni test was used to analyse two independent treatments at one time point. Differences were considered significant at values of P < 0.05. Statistical tests are specified in figure legends.

## Additional Information

**How to cite this article**: Labuz, D. *et al*. Distinct roles of exogenous opioid agonists and endogenous opioid peptides in the peripheral control of neuropathy-triggered heat pain. *Sci. Rep.* **6**, 32799; doi: 10.1038/srep32799 (2016).

## Supplementary Material

Supplementary Information
