# Supplementary material for: Distinct roles of exogenous opioid agonists and endogenous opioid peptides in the peripheral control of neuropathy-triggered heat pain
Source: Sci Rep. 2016 Sep 8;6:32799. doi: 10.1038/srep32799 (PMC5015056; doi:10.1038/srep32799)
Supplement: Supplementary Information [file srep32799-s1.pdf]

Supplementary Tables 1–5

Distinct roles of exogenous opioid agonists and endogenous opioid peptides in the peripheral control of neuropathy-triggered heat pain

Dominika Labuz, Melih Ö Celik, Andreas Zimmer & Halina Machelska

Supplementary Table 1. Detailed statistical evaluation for Figure 1.

Two-way repeated measures ANOVA

END

Wild-type CCI vs. END-KO CCI	Mechanical hypersensitivity
Genotype	$F_{1,11}$ = 0.260, P = 0.632
Time	$F_{9,45}$ = 113.379, P < 0.001
Genotype x Time	$F_{9,119}$ = 0.375, P = 0.94

Wild-type Sham vs. END-KO Sham	Mechanical hypersensitivity
Genotype	$F_{1,11}$ = 0.00843, P = 0.930
Time	$F_{9,45}$ = 0.561, P = 0.822
Genotype x Time	$F_{9,119}$ = 0.403, P = 0.927

Wild-type CCI vs. Wild-type Sham	Mechanical hypersensitivity
Treatment	$F_{1,11}$ = 248.755, P < 0.001
Time	$F_{9,45}$ = 7.725, P < 0.001
Treatment x Time	$F_{9,119}$ = 5.293, P < 0.001

END-KO CCI vs. END-KO Sham	Mechanical hypersensitivity
Treatment	$F_{1,11}$ = 521.547, P = 0.001
Time	$F_{9,45}$ = 8.299 , P < 0.001
Treatment x Time	$F_{9,119}$ = 6.346, P < 0.001

ENK

Wild-type CCI vs. PENK-KO CCI	Mechanical hypersensitivity
Genotype	$F_{1,15}$ = 5.888, P = 0.046
Time	$F_{9,63}$ = 161.671, P < 0.001
Genotype x Time	$F_{9,159}$ = 1.324, P = 0.243

Wild-type Sham vs. PENK-KO Sham	Mechanical hypersensitivity
Genotype	$F_{1,15}$ = 0.0372, P = 0.852
Time	$F_{9,63}$ = 0.897, P = 0.533
Genotype x Time	$F_{9,159}$ = 0.286, P = 0.976

PENK-KO CCI vs. PENK-KO Sham	Mechanical hypersensitivity
Treatment	$F_{1,15}$ = 265.887, P = 0.001
Time	$F_{9,63}$ = 18.645 , P < 0.001
Treatment x Time	$F_{9,159}$ = 11.544, P < 0.001

DYN

Wild-type CCI vs. PDYN-KO CCI	Mechanical hypersensitivity
Genotype	$F_{1,15}$ = 13.020, P = 0.009
Time	$F_{9,63}$ = 119.191, P < 0.001
Genotype x Time	$F_{9,159}$ = 3.676, P < 0.001

Wild-type Sham vs. PDYN-KO Sham	Mechanical hypersensitivity
Genotype	$F_{1,15}$ = 0.0516, P = 0.827
Time	$F_{9,63}$ = 0.658, P = 0.743
Genotype x Time	$F_{9,159}$ = 0.970, P = 0.970

Wild-type CCI vs. Wild-type Sham	Mechanical hypersensitivity
Treatment	$F_{1,15}$ = 862.763, P < 0.001
Time	$F_{9,63}$ = 18.126, P < 0.001
Treatment x Time	$F_{9,159}$ = 13.230, P < 0.001

PDYN-KO CCI vs. PDYN-KO Sham	Mechanical hypersensitivity
Treatment	$F_{1,15}$ = 687.568, P < 0.001
Time	$F_{9,63}$ = 10.837, P < 0.001
Treatment x Time	$F_{9,159}$ = 12.027, P < 0.001

Supplementary Table 2. Detailed statistical evaluation for Figure 3.

One-way ANOVA

Dose-dependency	
2 days	$F_{4,25} = 0.763$, $P = 0.559$
14 days	$F_{4,25} = 5.779$, $P = 0.002$

Two-way repeated measures ANOVA

Time-course	2 days	14 days
Treatment	$F_{1,11} = 0.0952$, $P = 0.770$	$F_{1,11} = 0.271$, $P = 0.625$
Time	$F_{6,30} = 235.345$, $P < 0.001$	$F_{6,30} = 207.957$, $P < 0.001$
Treatment x Time	$F_{6,83} = 4.301$, $P = 0.003$	$F_{6,83} = 0.481$, $P = 0.817$

Supplementary Table 3. Detailed statistical evaluation for Figure 4.

Two-way repeated measures ANOVA

END

Wild-type CCI vs. END-KO CCI	Heat hypersensitivity
Genotype<br>Time<br>Genotype x Time	$F_{1,11} = 0.000767$, $P = 0.979$<br>$F_{9,45} = 28.451$, $P < 0.001$<br>$F_{9,119} = 0.811$, $P = 0.608$

Wild-type Sham vs. END-KO Sham	Heat hypersensitivity
Genotype<br>Time<br>Genotype x Time	$F_{1,11} = 1.067$, $P = 0.349$<br>$F_{9,45} = 1.528$, $P = 0.167$<br>$F_{9,119} = 0.958$, $P = 0.487$

Wild-type CCI vs. Wild-type Sham	Heat hypersensitivity
Treatment<br>Time<br>Treatment x Time	$F_{1,11} = 2953.716$, $P < 0.001$<br>$F_{9,45} = 26.508$, $P < 0.001$<br>$F_{9,119} = 25.532$, $P < 0.001$

END-KO CCI vs. END-KO Sham	Heat hypersensitivity
Treatment<br>Time<br>Treatment x Time	$F_{1,11} = 849.558$, $P < 0.001$<br>$F_{9,45} = 3.835$, $P < 0.001$<br>$F_{9,119} = 4.280$, $P < 0.001$

ENK

Wild-type CCI vs. PENK-KO CCI	Heat hypersensitivity
Genotype<br>Time<br>Genotype x Time	$F_{1,15} = 0.822$, $P = 0.395$<br>$F_{9,63} = 3.957$, $P < 0.001$<br>$F_{9,159} = 1.161$, $P = 0.336$

Wild-type Sham vs. PENK-KO Sham	Heat hypersensitivity
Genotype<br>Time<br>Genotype x Time	$F_{1,15} = 1.086$, $P = 0.332$<br>$F_{9,63} = 0.655$, $P = 0.746$<br>$F_{9,159} = 0.939$, $P = 0.498$

Wild-type CCI vs. Wild-type Sham	Heat hypersensitivity
Treatment<br>Time<br>Treatment x Time	$F_{1,15} = 465.392$, $P < 0.001$<br>$F_{9,63} = 13.884$, $P < 0.001$<br>$F_{9,159} = 7.469$, $P < 0.001$

PENK-KO CCI vs. PENK-KO Sham	Heat hypersensitivity
Treatment<br>Time<br>Treatment x Time	$F_{1,15}= 859.433$, $P < 0.001$<br>$F_{9,63} = 15.013$, $P < 0.001$<br>$F_{9,159} = 28.905$, $P < 0.001$

DYN

Wild-type CCI vs. PDYN-KO CCI	Heat hypersensitivity
Genotype<br>Time<br>Genotype x Time	$F_{1,15} = 19.606$, $P = 0.003$<br>$F_{9,63} = 126.219$, $P < 0.001$<br>$F_{9,159} = 1.487$, $P = 0.172$

Wild-type Sham vs. PDYN-KO Sham	Heat hypersensitivity
Genotype<br>Time<br>Genotype x Time	$F_{1,15} = 0.580$, $P = 0.471$<br>$F_{9,63} = 0.560$, $P = 0.824$<br>$F_{9,159} = 0.942$, $P = 0.496$

Wild-type CCI vs. Wild-type Sham	Heat hypersensitivity
Treatment<br>Time<br>Treatment x Time	$F_{1,15} = 574.900$, $P < 0.001$<br>$F_{9,63} = 27.087$, $P < 0.001$<br>$F_{9,159} = 23.428$, $P < 0.001$

PDYN-KO CCI vs. PDYN-KO Sham	Heat hypersensitivity
Treatment<br>Time<br>Treatment x Time	$F_{1,15} = 983.712$, $P < 0.001$<br>$F_{9,63} = 27.213$, $P < 0.001$<br>$F_{9,159} = 16.321$, $P < 0.001$

Supplementary Table 4. Detailed statistical evaluation for Figure 5.

Two-way ANOVA

END

2 days, Wild-type vs. END-KO	DAMGO	DPDPE	U50,488H
Genotype	$F_{1,1}$ = 0.196, P = 0.662	$F_{1,1}$ = 2.142, P = 0.154	$F_{1,1}$ = 0.885, P = 0.355
Treatment	$F_{1,1}$ = 236.803, P < 0.001	$F_{1,1}$ = 499.141, P < 0.001	$F_{1,1}$ = 235.247, P < 0.001
Genotype x Treatment	$F_{1,28}$ = 0.388, P = 0.538	$F_{1,28}$ = 2.915, P = 0.099	$F_{1,28}$ = 1.210, P = 0.281

14 days, Wild-type vs. END-KO	DAMGO	DPDPE	U50,488H
Genotype	$F_{1,1}$ = 1.028, P = 0.319	$F_{1,1}$ = 4.950, P = 0.034	$F_{1,1}$ = 0.0361, P = 0.851
Treatment	$F_{1,1}$ = 525.486, P < 0.001	$F_{1,1}$ = 561.831, P < 0.001	$F_{1,1}$ = 939.403, P < 0.001
Genotype x Treatment	$F_{1,28}$ = 0.688, P = 0.414	$F_{1,28}$ = 0.0668, P = 0.798	$F_{1,28}$ = 6.798, P = 0.014

ENK

2 days, Wild-type vs. PENK-KO	DAMGO	DPDPE	U50,488H
Genotype	$F_{1,1}$ = 0.39, P = 0.537	$F_{1,1}$ = 1.7007, P = 0.201	$F_{1,1}$ = 2.057, P = 0.161
Treatment	$F_{1,1}$ = 185.122, P < 0.001	$F_{1,1}$ = 332.001, P < 0.001	$F_{1,1}$ = 169.433, P < 0.001
Genotype x Treatment	$F_{1,32}$ = 0.870, P = 0.358	$F_{1,32}$ = 0.857, P = 0.362	$F_{1,32}$ = 1.396, P = 0.246

14 days, Wild-type vs. PENK-KO	DAMGO	DPDPE	U50,488H
Genotype	$F_{1,1}$ = 1.745, P = 0.196	$F_{1,1}$ = 16.533, P < 0.001	$F_{1,1}$ = 3.055, P = 0.090
Treatment	$F_{1,1}$ = 228.196, P < 0.001	$F_{1,1}$ = 561.831, P < 0.001	$F_{1,1}$ = 512.984, P < 0.001
Genotype x Treatment	$F_{1,32}$ = 2.362, P = 0.134	$F_{1,31}$ = 0.222, P = 0.641	$F_{1,32}$ = 2.737, P = 0.108

DYN

2 days, Wild-type vs. PDYN-KO	DAMGO	DPDPE	U50,488H
Genotype	$F_{1,1}$= 0.112, P = 0.740	$F_{1,1}$ = 1.177, P = 0.286	$F_{1,1}$ = 1.436, P = 0.240
Treatment	$F_{1,1}$ = 149.543, P < 0.001	$F_{1,1}$= 228.984, P < 0.001	$F_{1,1}$ = 280.584, P < 0.001
Genotype x Treatment	$F_{1,32}$ = 0.415, P = 0.524	$F_{1,32}$ = 0.548, P = 0.465	$F_{1,32}$ = 0.733, P = 0.398

14 days, Wild-type vs. PDYN-KO	DAMGO	DPDPE	U50,488H
Genotype	$F_{1,1}$ = 1.868, P = 0.181	$F_{1,1}$ = 1.089, P = 0.305	$F_{1,1}$ = 0.0849, P = 0.773
Treatment	$F_{1,1}$ = 143.744, P < 0.001	$F_{1,1}$ = 163.491, P < 0.001	$F_{1,1}$ = 129.896, P < 0.001
Genotype x Treatment	$F_{1,32}$ = 0.912, P = 0.347	$F_{1,31}$ = 0.980, P = 0.330	$F_{1,32}$ = 4.678, P = 0.038

Supplementary Table 5. Detailed statistical evaluation for Figure 6.

One-way ANOVA

Mechanical hypersensitivity	END	ENK	DYN
2 days	$F_{3,28} = 32.731$, $P < 0.001$	$F_{3,28} = 24.521$, $P < 0.001$	$F_{3,28} = 63.054$, $P < 0.001$
14 days	$F_{3,28} = 19.319$, $P < 0.001$	$F_{3,28} = 56.751$, $P < 0.001$	$F_{3,28} = 44.390$, $P < 0.001$

Heat hypersensitivity	END	ENK	DYN
2 days	$F_{3,28} = 0.161$, $P = 0.922$	$F_{3,28} = 2.140$, $P = 0.118$	$F_{3,28} = 1.438$, $P = 0.253$
14 days	$F_{3,28} = 0.513$, $P = 0.677$	$F_{3,28} = 0.705$, $P = 0.557$	$F_{3,27} = 4.858$, $P = 0.008$
